# Schizophrenia and cell senescence candidate genes screening, machine learning, diagnostic models, and drug prediction

**DOI:** 10.3389/fpsyt.2023.1105987

**Published:** 2023-04-11

**Authors:** Yu Feng, Jing Shen, Jin He, Minyan Lu

**Affiliations:** ^1^The Affiliated Jiangsu Shengze Hospital of Nanjing Medical University, Suzhou, China; ^2^The University of New South Wales, Kensington, NSW, Australia; ^3^The University of Melbourne, Parkville, VIC, Australia

**Keywords:** schizophrenia, cell senescence, drug prediction, diagnostic model, immune infiltration, machine learning

## Abstract

**Background:**

Schizophrenia (SC) is one of the most common psychiatric diseases. Its potential pathogenic genes and effective treatment methods are still unclear. Cell senescence has been confirmed in mental diseases. A link exists between cellular senescence and immunity, and immune-related problems affect suicide rates in individuals suffering from schizophrenia. Therefore, the aims of this study were to identify candidate genes based on cell senescence that can affect the diagnosis and treatment of schizophrenia.

**Methods:**

Two data sets of schizophrenia were provided by the Gene Expression Omnibus (GEO) database, one was taken as training and the other as a validation group. The genes related to cell senescence were obtained from the CellAge database. DEGs were identified using the Limma package and weighted gene co-expression network analysis (WGCNA). The function enrichment analysis was conducted, followed by machine learning-based identification for least absolute shrinking and selection operators (LASSO) regression. Random Forest were used to identify candidate immune-related central genes and establish artificial neural networks for verification of the candidate genes. The receiver operating characteristic curve (ROC curve) was used for the diagnosis of schizophrenia. Immune cell infiltrates were constructed to study immune cell dysregulation in schizophrenia, and relevant drugs with candidate genes were collected from the DrugBank database.

**Results:**

Thirteen co-expression modules were screened for schizophrenia, of which 124 were the most relevant genes.

There were 23 intersected genes of schizophrenia (including DEGs and the cellular senescence-related genes), and through machine learning six candidate genes were finally screened out. The diagnostic value was evaluated using the ROC curve data. Based on these results it was confirmed that these candidate genes have high diagnostic value.

Two drugs related to candidate genes, Fostamatinib and Ritodine, were collected from the DrugBanks database.

**Conclusion:**

Six potential candidate genes (SFN, KDM5B, MYLK, IRF3, IRF7, and ID1) had been identified, all of which had diagnostic significance. Fostamatinib might be a drug choice for patients with schizophrenia to develop immune thrombocytopenic purpura (ITP) after treatment, providing effective evidence for the pathogenesis and drug treatment of schizophrenia.

## 1. Introduction

Schizophrenia is a psychotic disorder characterized by hallucinations, delusions, overthinking, perception and behavioral disorders (1). More than half of the patients have serious complications including mental and physical problems, making it one of the major causes of disability worldwide (2). The life span of patients is about 20% less than that of normal people, and up to 40% of deaths are attributed to suicide (3). Suicidal behavior is complex and is caused by a variety of factors, one of which involves immune system dysregulation. This has been implicated in the pathophysiology of suicidal behavior (4).

Growing evidence has revealed that patients with severe mental illness might have accelerated senescence. Especially in elderly patients who are prone to systemic diseases and increased mortality (5). Cell senescence is a process that primarily removes unwanted cells by inducing tissue remodeling (6). Biological senescence is marked by cell senescence, and an important mark of the latter is the length of the telomere or more specifically the telomere length of the white blood cells (7, 8). Telomere shortening may lead to increased cell death and reduced regeneration of stem cells and progenitor cells which will damage the process of cell replacement and repair, leading to rapid disease development (9). Senescence immune cells can secrete proinflammatory cytokines, which may also lead to a vicious cycle of inflammation, oxidative stress and telomere shortening (10). According to previous studies, non-remissioning schizophrenia patients had shorter relative telomere lengths, suggesting that they may share biological pathways with other neurodegenerative diseases characterized by increased cellular senescence (11).

The development of biomedicine has been tremendously aided by breakthrough advancements in microarray technology and bioinformatics. Public databases have a lot of high-throughput data, which greatly helps in revealing disease pathogenesis and identifying potential targets for drug designing (12). However, the applications of these techniques are very adequate to combat schizophrenia. This study aims to fill the gap and provide more effective choices for drug selection.

## 2. Materials and methods

### 2.1. Materials

**Figure 1:**
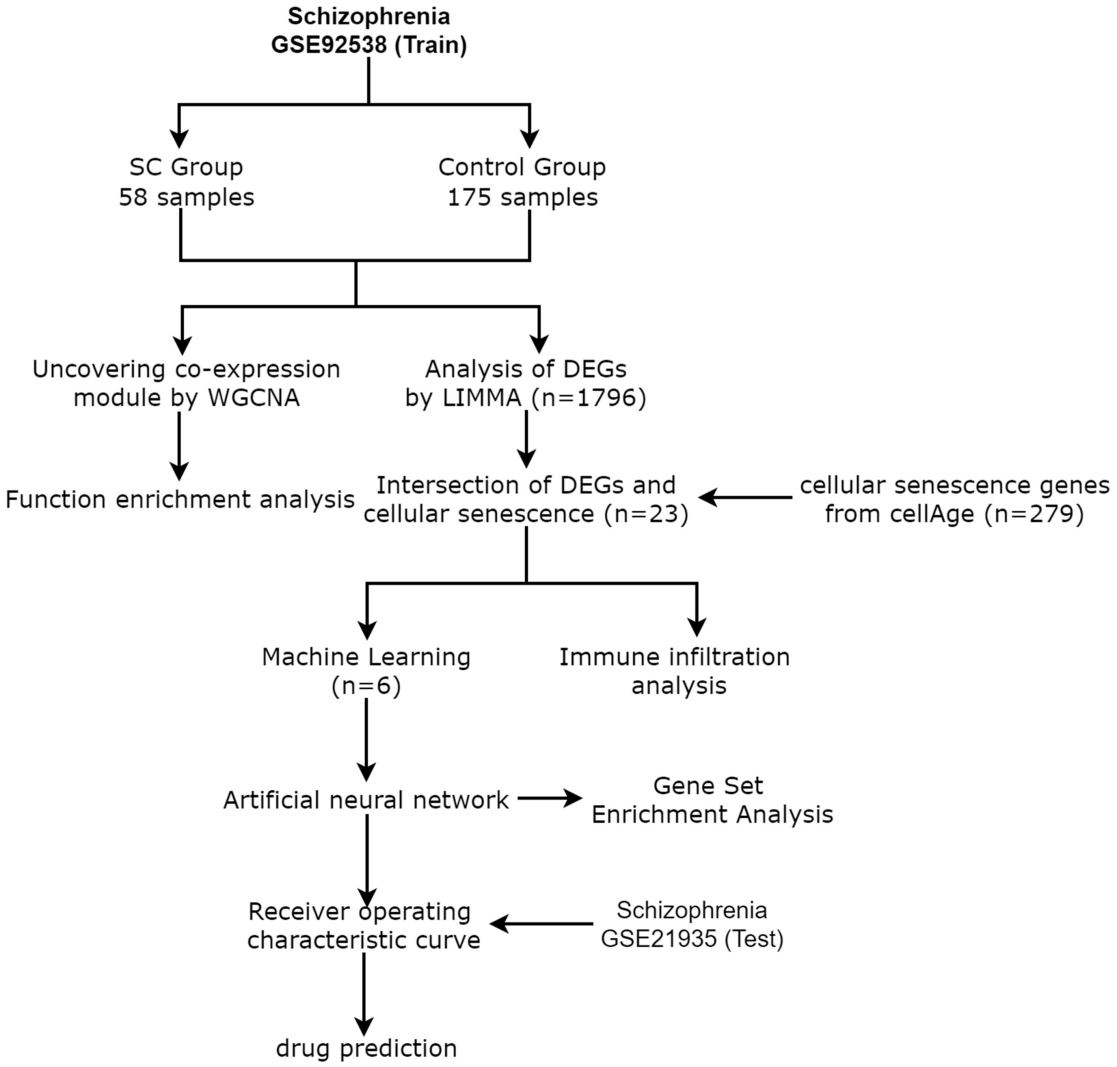
Flow Chart.

From the GEO database,^1^ the schizophrenia data set GSE92538 was selected as the training group and GSE21935 as the test group (13). CellAge database ^2^ was used to retrieve 279 cellular senescence-related genes (14). Figure 1 shows the process flow and the complete information dataset is given in Table 1.

**Table 1:** Detailed data set information.

GSE series	Type	Sample size		Platform
*Schizophrenia(Train) Control*				
GSE92538	mRNA	58	175	GPL10526
*Schizophrenia(Test) Control*				
GSE21935	mRNA	23	19	GPL570
*Cellular senescence genes*				
CellAge	mRNA	279		cellAge

### 2.2. Modular gene selection and weighted gene co-expression network analysis

Gene expression profile was used (15) to calculate the MAD (Medium Absolute Development) of all genes in the GSE92538 dataset, and the first 25% of the genes with the least MAD were removed. To eliminate the genes and sample outliers, the GoodSamplesGenes method of the WGCNA package of R software was utilized and further construction of a scale-free co-expression network was also done by WGCNA (the sensitivity was kept 3). Furthermore, modules with a distance of less than 0.25 were combined, and as a result, 13 co-expression modules were obtained. It was important to note that the grey module was regarded as a gene set that was not compatible with any module.

### 2.3. Gene function enrichment analysis

Gene functional enrichment analysis of the above-processed module genes was performed to understand the main expression forms of schizophrenia genes. For gene set functional enrichment analysis, KEGG rest API () was used to acquire gene annotation of the KEGG pathway. Additionally, GO annotations for genes were used from “R” (version 3.1.0) (org.Hs.eg.db) (16). To acquire the findings of the gene set enrichment, they were mapped onto the background set through ClusterProfiler (version 3.14.3) of “R” (17). The GSEA software (version 3.0) was downloaded for GSEA from its website ^3^ (18). To assess the associated pathways and molecular mechanisms, the samples were separated into two groups and the subsets were downloaded from the Molecular Signatures Database (c2.cp.kegg.v7.4.symbols.gmt) (19). A minimum gene set of 5 and a maximum gene set of 5,000 was determined on the basis of gene expression profiles and phenotypic grouping. *p* value <0.05 and FDR of <0.1 were considered statistically significant.

### 2.4. Identification and screening of DEGs related to cell senescence

The Limma package (linear models for microarray data) (version 3.40.6) (20) of the R software was used for screening DEGs between comparison groups and control groups. In this study, | log2 multiple change (FC) | > 0.5 and p value <0.05 were selected as the criteria for the Limma package to identify differential gene expression (DGE). The related genes were cross-screened to schizophrenia DEG and cell senescence through Venn diagrams (21), and heat maps and volcano plots of cell senescence-related DEG were visualized through sangerBox (22).

### 2.5. Screening of senescence-related characteristic genes of schizophrenic cells by machine learning

In the current study, the gene expression data, its survival time, and survival status were integrated using glmnet (23) and RandomForest (24) of the R software, and regression analysis were carried out using the Random Forest and lasso-cox methods. In addition, to optimize the model, 10-fold cross-validation was set. The neuralnet (25) of “R” was used to construct an artificial neural network for the feature genes obtained by the above methods, to build a high-precision diagnostic model. Further, pROC (26) of “R” was used to conduct ROC analysis to obtain AUC, and the CI tool of the pROC was utilized for the evaluation of AUC and CI (confidence interval) to obtain the final results. The sangerBox software was used for visualization and observation of the expressed characteristic genes in the training set (GSE92538) and test group (GSE21935).

### 2.6. Immune infiltration analysis

The corrplot of “R” (27) was used to create the heat maps related to invasive immune cells, and Cibersort (28) was utilized to examine the infiltration of immune cells.

### 2.7. Drug prediction

The DrugBank database, a free access and comprehensive online database with information about drugs and their targets were used to retrieve the drug-targeting central genes (29). The drugs related to the characteristic genes were recorded and visualized for further correlation analysis.

## 3. Results

### 3.1. Modular gene selection and weighted gene co-expression network analysis

**Figure 2:**
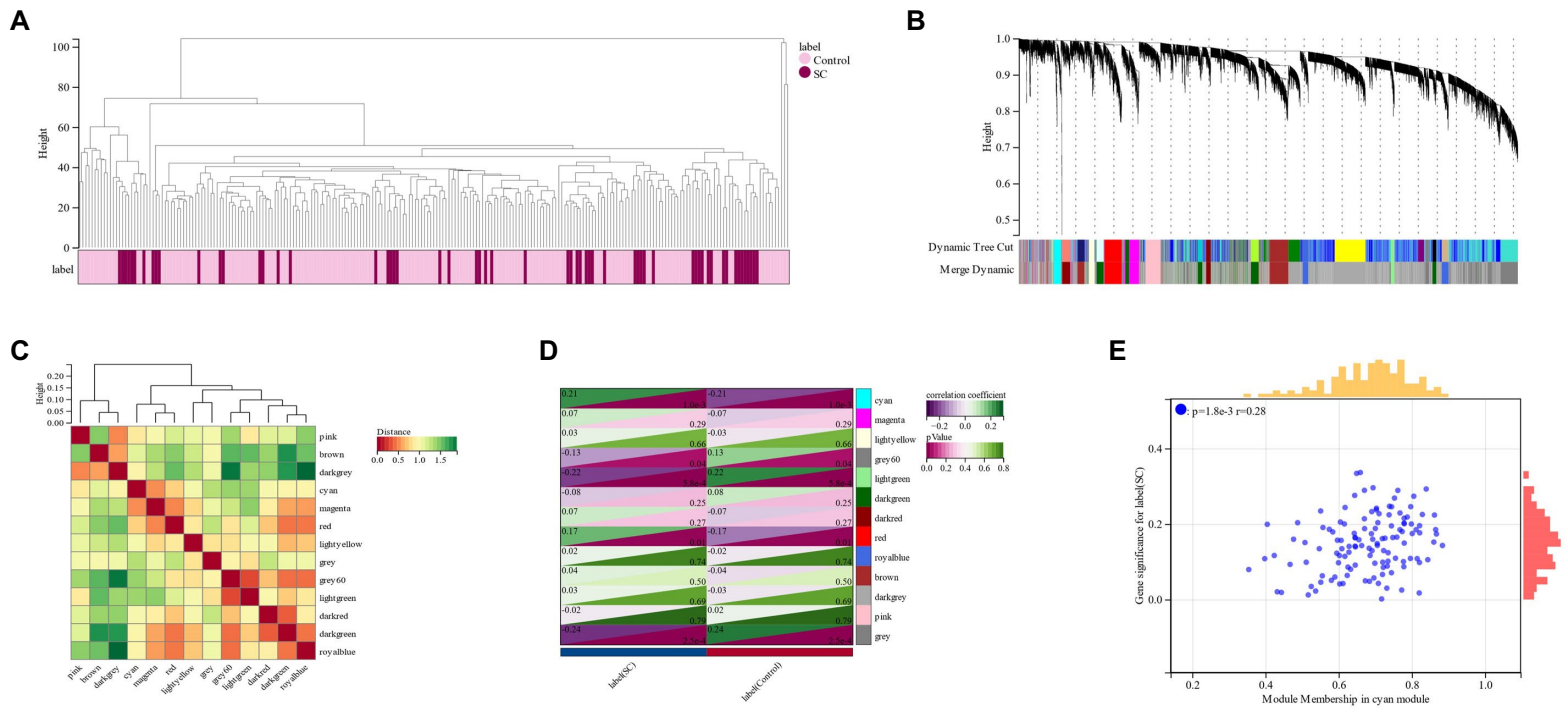
**(A)** The Clustered tree diagram of schizophrenia and its control group; **(B,C)**: Schizophrenia gene co-expression module (GCM); **(D)**: Correlation between schizophrenia and GCM; **(E)**: The correlation between module members and gene significance in the Cyan module of schizophrenia.

A cluster tree diagram of schizophrenia and the control group was created using the soft threshold *β* = 6 (Figure 2A). As a result, 13 GCM (gene co-expression modules) were constructed (Figures 2B,C), and the relationship between GCM and schizophrenia was demonstrated (Figure 2D). Cyan module (124 genes) was found to have the highest correlation with schizophrenia (correlation coefficient = 0.21, *p* = 1.0e-3). The correlation between module members and gene significance in the cyan module of schizophrenia was also calculated, and a significant positive correlation was observed (*r* = 0.28) (Figure 2E).

### 3.2. Functional enrichment analysis of schizophrenia

**Figure 3:**
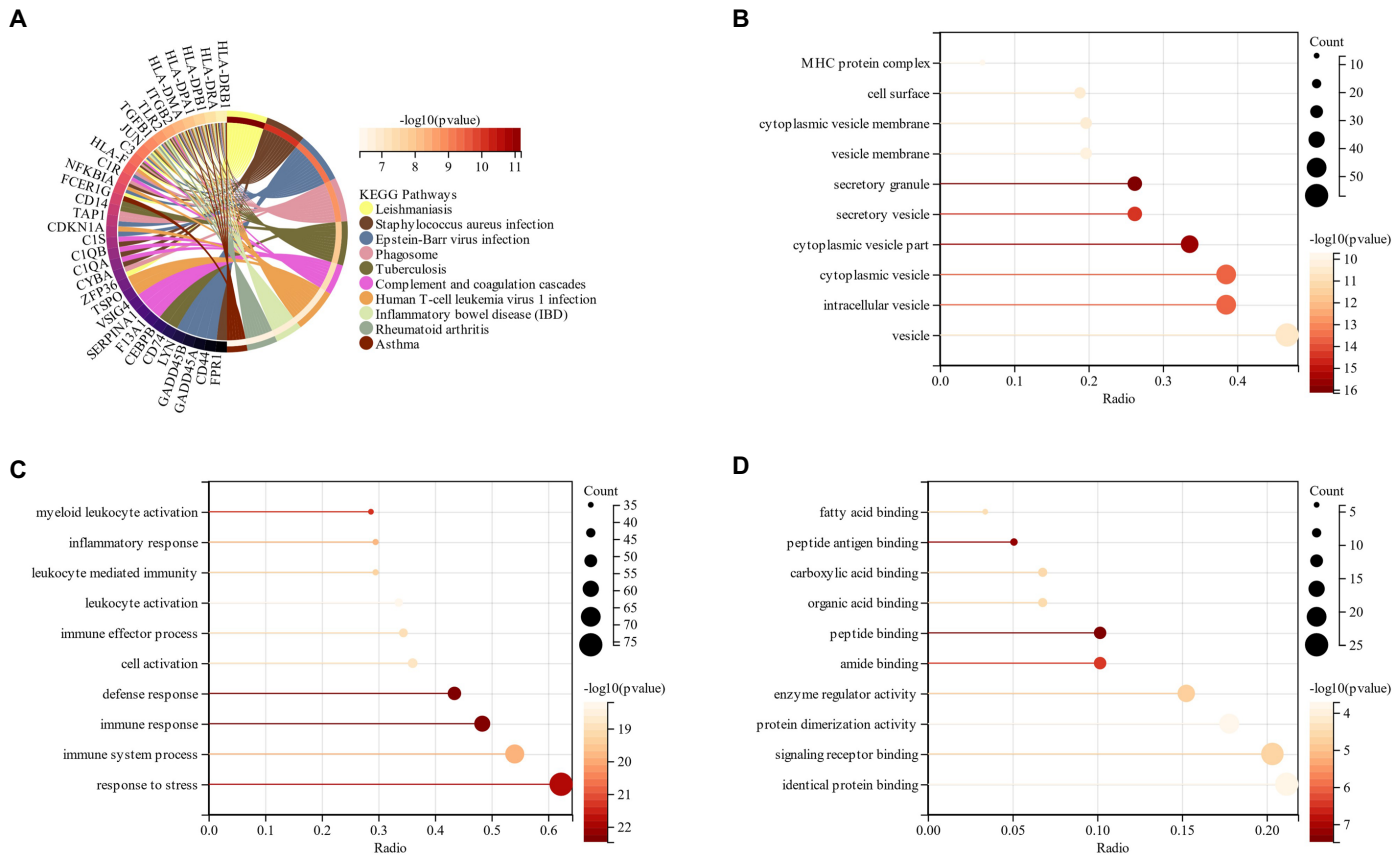
**(A)** KEGG analysis of schizophrenia CGs; **(B)**: GO analysis of cell composition (CC) of schizophrenia CGs; **(C)**: GO analysis of the biological process (BP) of schizophrenia CGs; **(D)**: GO analysis molecular function (MF) of schizophrenia CGs.

The processed schizophrenia module genes were subjected to functional enrichment analysis. KEGG analysis showed that CSs were mostly present in the “Epstein–Barr virus infection,” “Phagosome” and “Tuberculosis” pathways (Figure 3A). The GO analysis revealed that CGs were primarily found in “vesicle,” “extracellular region,” and “cyclomatic vesicle” sites of cell components (CC; Figure 3B). The key biological processes (BP) of CGs included the immune system process, response to stress and response to an organic substance (Figure 3C). Based on the molecular function (MF) it was revealed that the key components in CGs were “signaling receiver binding” and “identity protein binding” (Figure 3D).

### 3.3. Screening of differentially expressed genes in schizophrenia and identification of cell senescence-related DEG

**Figure 4:**
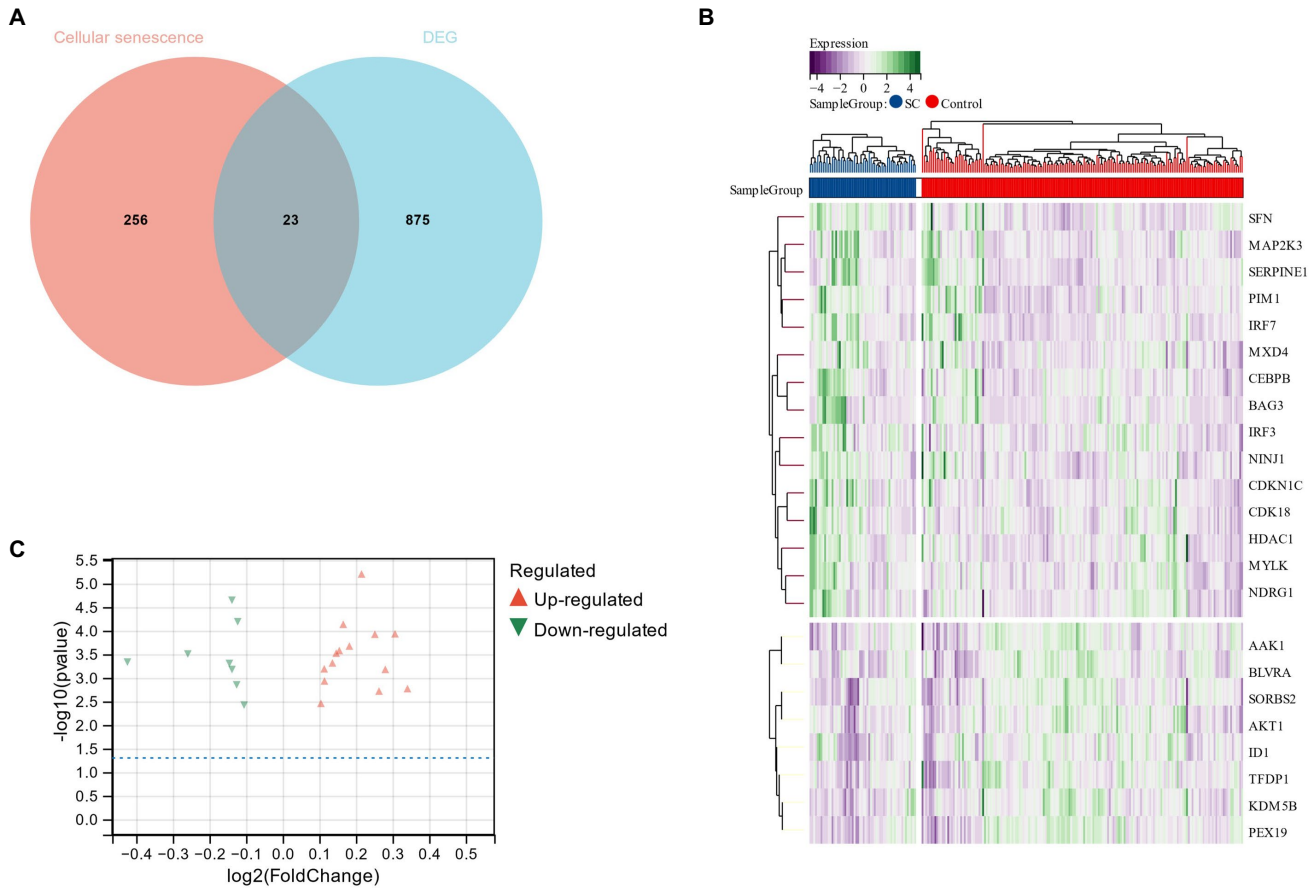
**(A)** Venn diagram for cross-screening of genes related to schizophrenia and cell senescence; **(B,C)**: Heat map and volcano plot of cell senescence DEG.

Using the Limma package, 1796 DEGs were identified in the schizophrenia data set (GSE92538), of which 898 were up-regulated and 898 were down-regulated. Schizophrenia DEGs and cell senescence-related genes were cross-screened through Venn diagrams to obtain 23 cell senescence DEGs (Figure 4A). In addition, relevant heat maps and volcano plots were drawn, including the 15 up-regulated and 8 down-regulated genes (Figures 4B,C).

### 3.4. Functional enrichment analysis of intersected genes related to schizophrenia and cell senescence

**Figure 5:**
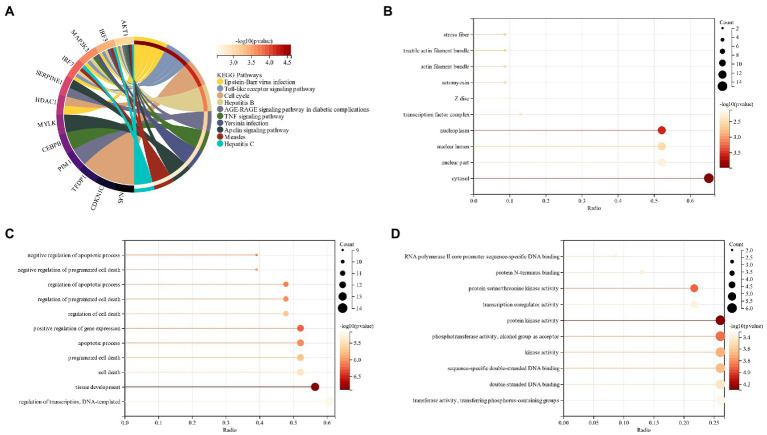
**(A)** KEGG analysis of intersected genes related to schizophrenia and cell senescence; **(B)**: GO analysis of the intersected genes of schizophrenia and cell senescence (CC); **(C)**: GO analysis of the biological process (BP) of intersected genes related to schizophrenia and cell senescence; **(D)**: GO Analysis of Molecular Function (MF) of intersected genes related to schizophrenia and cell senescence.

The functional enrichment of genes related to schizophrenia and cell senescence was analyzed. KEGG analysis revealed that CSs were most abundant in the “Epstein–Barr virus infection,” “Toll-like receptor signaling” and “Hepatitis B” pathways (Figure 5A). GO analysis revealed that in terms of CC, CGs were mainly located in “cyclosol,” “nucleoplasma” and “nuclear lumen” (Figure 5B). “Tissue formation,” “positive regulation of gene expression,” and “adaptive process” were among the major biological processes (BP) of CGs (Figure 5C). Based on molecular function (MF), the most significant components of CGs were “protein kinase activity,” “kinase activity,” and “double translated DNA binding” (Figure 5D). By comparing the functional enrichment analysis of schizophrenia, it was revealed that both of them mainly occurred in the pathway related to virus and immunity, which also verified the correlation between them.

### 3.5. Screening candidate genes through machine learning and construction of an artificial neural network

**Figure 6:**
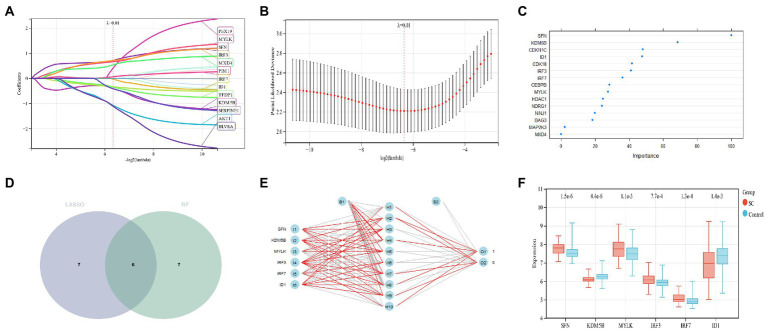
**(A,B)** Candidate gene screening of LASSO regression; **(C)**: Screening of RF candidate genes; **(D)**: Two kinds of machine learning intersected genes; **(E)**: Artificial neural network construction; **(F)**: Expression profile analysis of six candidate genes.

LASSO regression analysis was used for the screening of candidate genes and 13 potential biomarkers were identified from these results (Figures 6A,B). Followed by the RF regression analysis for the screening of candidate genes and 13 potential candidate biomarkers were displayed from it (Figure 6C). Then the results from the two aforementioned techniques were cross-analyzed, and finally, six candidate genes (SFN, KDM5B, MYLK, IRF3, IRF7, and ID1) were obtained (Figure 6D). A neutral network was constructed using these six genes, and the results of these confirmed that the six candidate genes could well differentiate the schizophrenia samples from the control samples (Figure 6E). The expression profile analysis of these six genes was evaluated, and results revealed that the candidate genes were considerably different between the control group and the schizophrenia group (Figure 6F).

### 3.6. Construction and verification of diagnostic model

**Figure 7:**
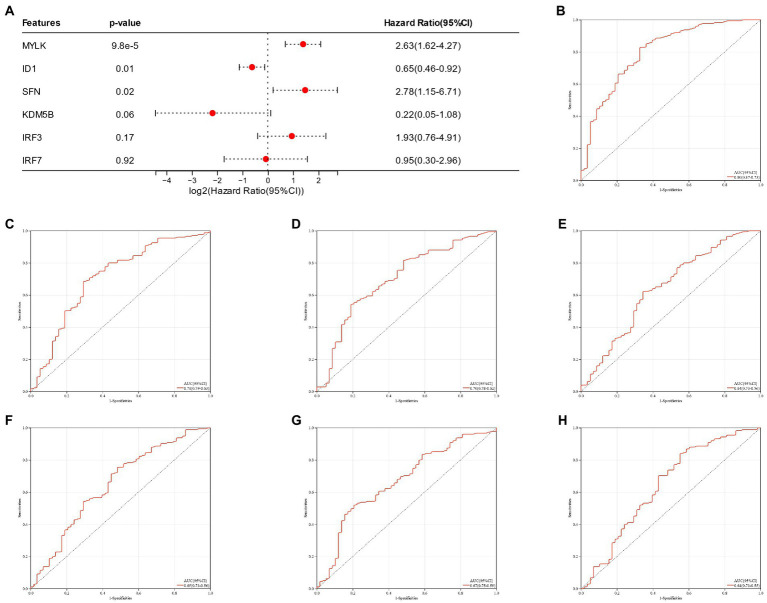
**(A)** Nomogram of candidate genes in the training group; **(B–H)**: Nomogram of the training group and ROC curves of candidate genes.

**Figure 8:**
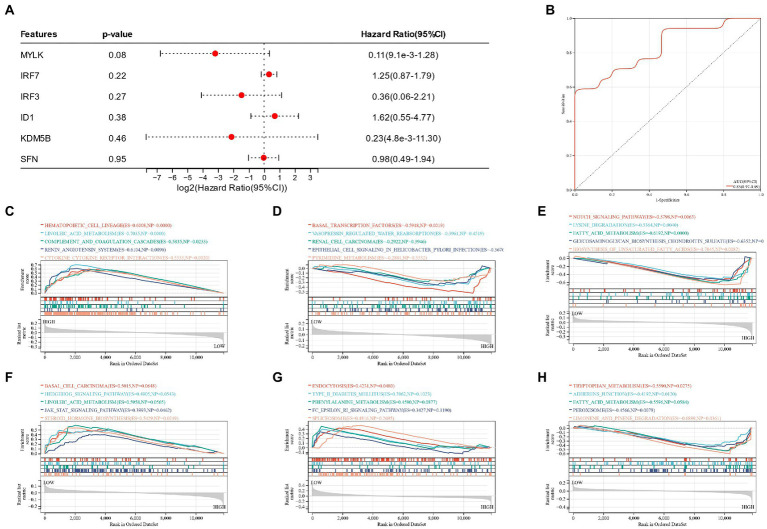
**(A)** Nomogram of candidate genes in the test group; **(B)**: ROC curves of nomogram of the test group; **(C**–**H)**: GSEA reveals the enrichment pathway of candidate genes.

A nomogram of six potential candidate genes was established (Figure 7A), the CI (95%) and AUC of each item were analyzed, and ROC curves were plotted to determine the specificity and sensitivity of each gene. Nomogram (AUC 0.80, CI 0.87–0.73), SFN (AUC 0.71, CI 0.79–0.63), KDM5B (AUC 0.70, CI 0.78–0.62), MYLK (AUC 0.64, CI 0.73–0.56), IRF3 (AUC 0.65, CI 0.73–0.56), IRF7 (AUC 0.67, CI 0.75–0.59), ID1 (AUC 0.64, CI 0.73–0.55; Figures 7B–H). These findings demonstrated the high diagnostic value of each candidate gene for metabolic syndrome and bipolar illness. In order to further verify the model, a nomograph of the candidate genes in the test group (GSE21935) and ROC curves (AUC 0.83, CI 0.97–0.69) was constructed (Figures 8A,B). The results of the test group showed that the model had certain significance for the diagnosis of schizophrenia. Moreover, the gene selection enrichment analysis (GSEA) on six candidate genes showed its correlation with metabolism (Linoleic acid metabolism, Tryptophan metabolism, Phenylalanine metabolism) (Figures 8C–H).

### 3.7. Analysis of immune cell infiltration

**Figure 9:**
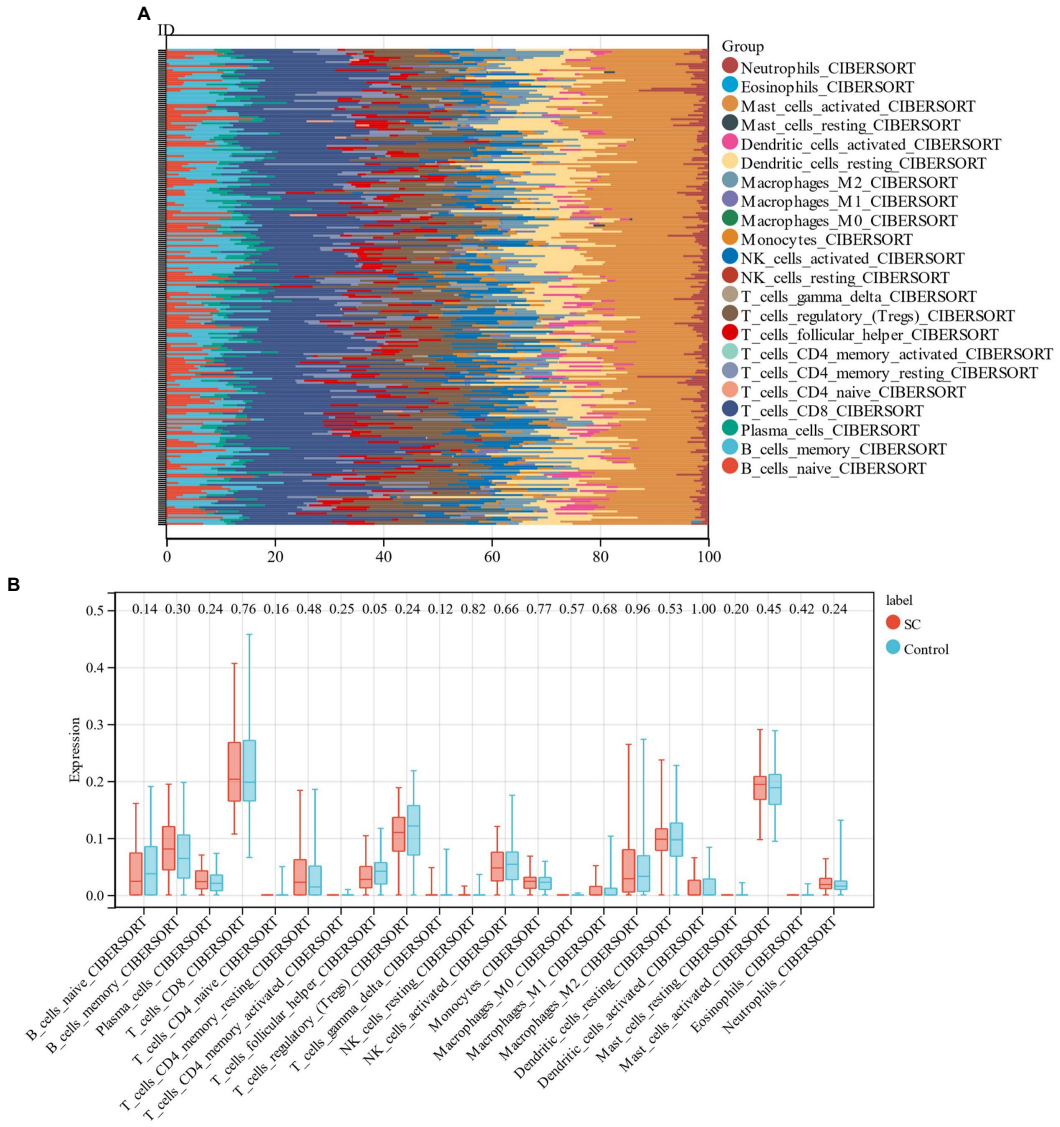
**(A)** Relative percentage of 22 immune cells in each sample; **(B)**: The difference in immune infiltration between schizophrenia samples and control samples.

In the current study, the CIBERSORT algorithm was used to assess the proportion of 22 immune cells in schizophrenia samples and control samples in the training group (GSE92,538; Figure 9A). Followed by the comparison of the immunocyte infiltration of schizophrenic and control samples in the box plot (Figure 9B). The results revealed that the levels of plasma cells, memory B cells, and M2 macrophages in schizophrenic patients were higher, but the levels of regulatory T cells, CD8+ T cells, and follicular helper T cells were lower.

### 3.8. Prediction of related drugs

**Figure 10:**
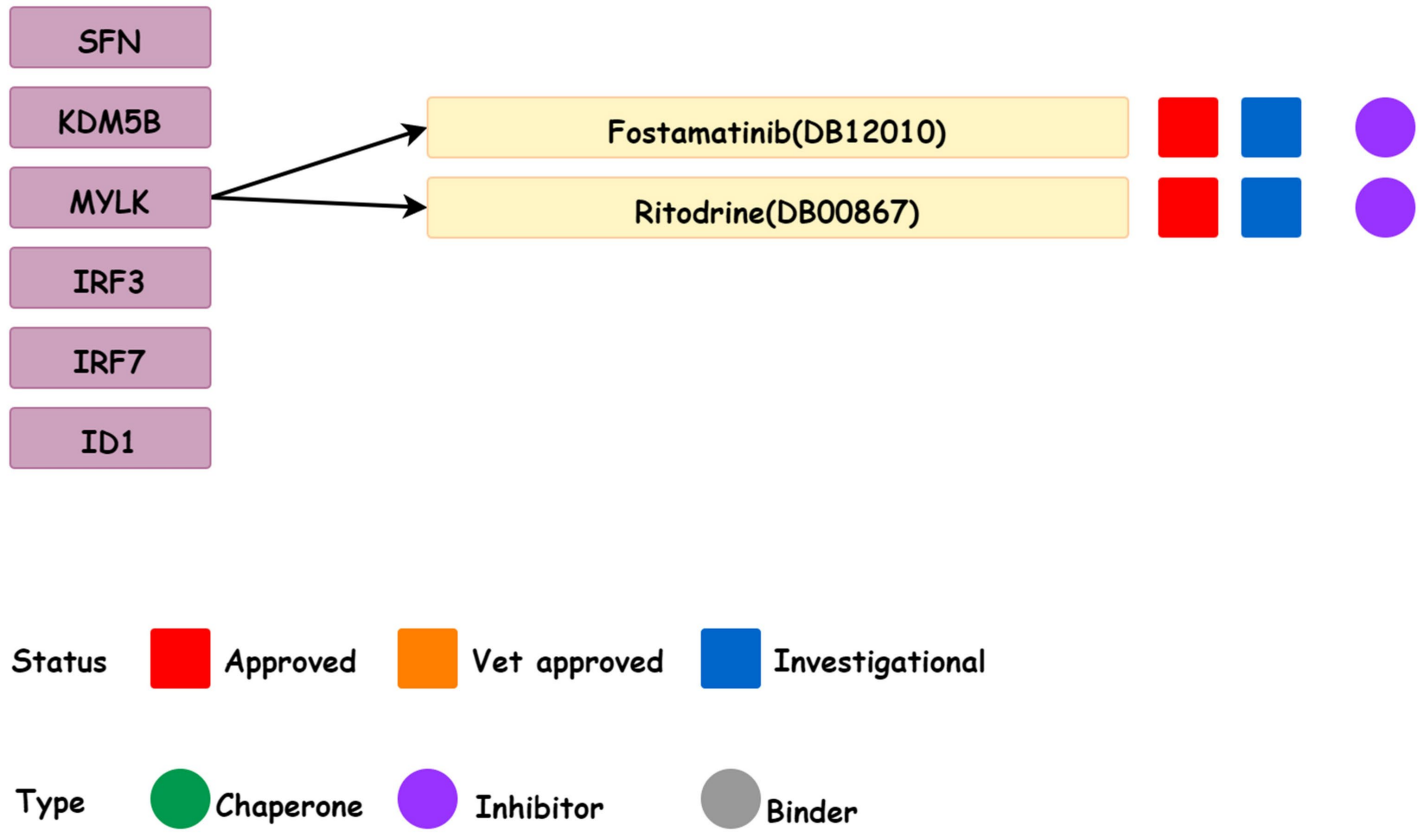
Drugs for the six candidate genes obtained from the DrugBank database.

DrugBank database was used to collect data on two drugs (Fostamatinib and Ritodine) that target six candidate genes (Figure 10) and they were the MYLK inhibitors. Fostamatinib was used for the treatment of immune thrombocytopenic purpura (ITP). While Ritodine was an adrenergic β receptor agonist used for the treatment of premature birth.

## 4. Discussion

Through KEGG analysis, we found that schizophrenia-related genes are associated with epstein–barr virus infection, and after analyzing the intersection of schizophrenia-associated genes and cell aging-associated genes, we concluded that these candidate genes are also significantly correlated with epstein–barr virus infection. Accordingly, we believe that schizophrenia is associated with immunity. Then we performed immune infiltration analysis and found that there are significant differences in follicular helper T cells between schizophrenia patients and controls. Furthermore, we established a schizophrenia diagnostic model based on genes associated with cellular senescence, which contain 6 candidate genes (SFN, KDM5B, MYLK, IRF3, IRF7, ID1).

Schizophrenia is a serious chronic mental disorder which is regarded as an accelerated cell aging syndrome, characterized by the early onset of cardiovascular disease leading to premature death (30). However, other complications are potentially important confounding factors in explaining cell senescence in mental diseases. Numerous psychiatric diagnoses have high complications with secondary psychiatric diseases, such as drug abuse, various anxiety disorders and post-traumatic stress disorder (31). Therefore, this study aims to find cell senescence genes related to single schizophrenia.

Among the six candidate genes screened in this study, IRF3 has been proven to possess differences (32). An interferon regulatory transcription factor (IRF) is encoded by this gene and upon serine/threonine phosphorylation, the encoded protein transforms into an active cytoplasmic version that binds to CREBBP. The transcription of the interferons alpha and beta as well as other interferon-induced genes activates when this complex translocates to the nucleus. This protein is crucial for the innate immune response to DNA and RNA viruses (33). IRF7 of the IRF family has also been proved to be related to schizophrenia (34). In a study of experimental mice, it was found that peripubertal viral-like challenge and isolation cause overlapping with apparent effects on behavior while constantly increasing brain IRF7 expression (35). It has been reported by Bashyer et al., that KDM5B is related to autism spectrum disorder (ASD) and other neurological diseases (36). This gene encodes a histone demethylase that is specific to lysine residues and belongs to the Jumanji/ARID domain-containing family. The encoded protein has the ability to demethylate the di-, tri-, and monomethylated lysine 4 of histone H3. This protein is overexpressed in some cancer cells and is mainly involved in the transcriptional regulation of a few tumor suppressor genes. Additionally, this protein may contribute to DNA repair and genomic stability (37).

It was confirmed from the DrugBank database (38) that Fostamatinib despite being a spleen tyrosine kinase inhibitor is also used in the treatment of chronic immune thrombocytopenia after other treatments. Numerous studies found the presence of ITP in schizophrenia patients after drug treatment (39, 40) and Fostamatinib is a strong inhibitor of MYLK. The findings of the current study suggest that the MYLK expression in schizophrenic patients is higher than that in the control group through gene expression profile. Therefore, Fostamatinib can be considered for combined treatment when ITP occurs in schizophrenic patients. Furthermore, we found that buprenorphine was an effective, well-tolerated, and safe option for reducing depressive symptoms, severe suicidal ideation, and nonsuicidal self-injury, even in immune-related disorders (41).

Shortcomings of this study: due to the lack of corresponding clinical research tools, no further confirmation with wet lab tests were carried out. The analysis could not be conducted in combination with clinical information. Moreover, due to the small number of data sets, peripheral blood-related validation could not be carried out.

## 5. Conclusion

Six candidate genes (SFN, KDM5B, MYLK, IRF3, IRF7, and ID1) were identified with diagnostic significance for schizophrenia. Fostamatinib may become a drug choice for schizophrenic patients that develop immune thrombocytopenic purpura (ITP) after treatment, providing effective evidence for the selection of schizophrenia pathogenesis and drug treatment.

## Data availability statement

The original contributions presented in the study are included in the article/supplementary material, further inquiries can be directed to the corresponding author.

## Author contributions

YF and JS wrote the main manuscript text. ML and JH provide modification assistance, and all authors reviewed the manuscript. All authors contributed to the article and approved the submitted version.

## Conflict of interest

The authors declare that the research was conducted in the absence of any commercial or financial relationships that could be construed as a potential conflict of interest.

## Publisher’s note

All claims expressed in this article are solely those of the authors and do not necessarily represent those of their affiliated organizations, or those of the publisher, the editors and the reviewers. Any product that may be evaluated in this article, or claim that may be made by its manufacturer, is not guaranteed or endorsed by the publisher.

## Footnotes

^1^ https://www.ncbi.nlm.nih.gov/geo/

^2^ https://genomics.senescence.info/cells/

^3^ http://software.broadinstitute.org/gsea/index.jsp
